# Challenges and strategies to improve recruitment in international, multi-centre research studies in locally recurrent rectal cancer: experience from the Locally Recurrent Rectal Cancer—Quality of Life (LRRC-QoL) study

**DOI:** 10.1186/s13063-025-09264-0

**Published:** 2026-04-25

**Authors:** Niamh McKigney, Julie Croft, Galina Velikova, Julia M. Brown, Deena P. Harji

**Affiliations:** 1 Clinical Trials Research Unit, Leeds Institute of Clinical Trials Research, University of Leeds, Worsley Building, Clarendon Way, Leeds, LS2 9NL UK; 2 Leeds Institute of Medical Research at St. James’s, University of Leeds, Leeds, UK; 3 Leeds Cancer Centre, St. James’s University Hospital, Leeds, UK; 4 Department of Colorectal Surgery, Manchester University NHS Foundation Trust, Manchester, UK

**Keywords:** Locally recurrent rectal cancer, Advanced malignancy, Recruitment challenges, International

## Abstract

**Background:**

Locally recurrent rectal cancer (LRRC) is an emerging area for research; however, it represents significant challenges as a relatively rare form of advanced pelvic malignancy, from both a recruitment and study setup and delivery perspective. To date, there have been relatively few published trials in this setting. High-quality, multi-centre, prospective studies could offer helpful insights regarding the challenges associated with delivering studies in rare disease settings such as LRRC, and how to effectively address them.

**Methods:**

The Locally Recurrent Rectal Cancer—Quality of Life (LRRC-QoL) study is an international, multi-centre, mixed-methods study of health-related quality of life (HrQoL) in LRRC. The International Surgical Trials Toolkit was utilised as a guideline in navigating site setup processes and to describe the challenges encountered during this study. A modified Quintet Recruitment Intervention (QRI) was used as a framework to identify recruitment challenges and drive improvements.

**Results:**

Overall, 227 patients were recruited to the LRRC-QoL study across 14 countries. Significant challenges were encountered during site setup, including issues related to legal agreements which were further complicated by Brexit, expenses related to translation, and requirements for multiple ethical approvals. Delays during study setup and recruitment challenges occurred due to the COVID-19 pandemic. Several strategies were identified through the modified QRI with a positive impact on recruitment. Recruitment pathways were refined to a more streamlined, centralised approach, facilitated by verbal consent to contact. Recruitment rates also improved with the introduction of multiple options for participation, including traditional paper-based methods, online, and via telephone. Patient information leaflets were refined following patient and public involvement (PPI) work.

**Conclusions:**

Several approaches identified during the LRRC-QoL study should be considered in the development of future studies and trials recruiting patients with LRRC. These include undertaking PPI during study development, identifying flexible recruitment strategies which complement sites’ existing clinical processes, and partnering with existing collaborative networks.

Study registration

The LRRC-QoL study registration reference: ISRCTN13692671 (https://doi.org/10.1186/ISRCTN13692671).

## Background

Locally recurrent rectal cancer (LRRC) is an important area for research within the global field of colorectal cancer surgery, due to its significant impact on patients [1], and on healthcare services, given the complexity of treatment decisions [2–4], and financial ramifications of surgical management [5]. Its standing as a relatively rare disease state presents additional challenges for researchers in their approach to study design and delivery; international collaboration takes on crucial importance, where shared experience is essential to accruing greater understanding. This has been demonstrated in LRRC through initiatives such as the PelvEx Collaborative, where centres have pooled their outcome data, leading to a significant improvement in outcome reporting in patients undergoing exenterative surgery [6]. However, published evidence from clinical trials in this setting remains limited.

Recruiting patients with advanced or recurrent malignancy to research studies presents distinct challenges, with reasons for non-participation including morbidity, being too unwell to participate, experiencing severe distress, or having other competing priorities [7]. Previous prospective studies in patients with advanced or recurrent rectal cancer have also illustrated the challenges of maintaining response rates during prospective quality of life (QoL) follow-up [8–10]. Recruitment specifically to QoL studies in this group may be further impaired by a perception that QoL studies include topics that are considered sensitive or personal, such as sexual function [11]. International, multi-centre recruitment is essential in rare disease settings; however, this presents additional challenges, including the diverse regulatory approvals required across different countries and at different sites, the negotiation of contracts for site setup, navigation of different time zones, and language barriers [12, 13]. Though randomised controlled trials (RCTs) will always represent the gold standard, the evidence and experiences of delivering non-randomised studies could provide helpful insights in overcoming challenges associated with delivering studies in rare disease settings such as LRRC.

The Locally Recurrent Rectal Cancer—Quality of Life (LRRC-QoL) study is an international, multi-centre, mixed-methods study of health-related quality of life (HrQoL) in LRRC consisting of two workstreams. The first workstream adapted the LRRC-QoL measure for use in several countries. Workstream II is a longitudinal, prospective observational cohort study of HrQoL in LRRC from baseline up to 12 months. Data from both workstreams I and II contributed to the international validation of the LRRC-QoL. This manuscript will outline some of the challenges experienced during the delivery of the LRRC-QoL study and the strategies implemented to overcome them, with a view to building on existing resources available to researchers working in rare forms of malignancy and specifically LRRC.

## Methods

Two resources informed the approaches which are described in this manuscript and were implemented during the delivery of the LRRC-QoL study. These were the International Surgical Trials Toolkit [14], which provided guidance in navigating site setup processes, and the Quintet Recruitment Intervention [15], which was used as a framework for the methods used to identify and address recruitment issues.

### The international surgical trials toolkit

The International Surgical Trials Toolkit (ISTT) was developed by the University of Leeds Clinical Trials Research Unit (CTRU) to improve the efficiency of the setup and conduct of international surgical trials and contains key areas for consideration in relation to study design and implementation [14]. These include sponsorship, finance, contracts, insurance, research governance, protocol, monitoring, trial supplies, data collection, sample collection, health economics, and data ownership and publication.

The ISTT was referred to throughout the process of setting up and running the LRRC-QoL study, particularly in relation to finances, translation, and contracts.

### Modified quintet recruitment intervention

The Quintet Recruitment Intervention (QRI) was developed to optimise recruitment to RCTs [15] and has demonstrated its effectiveness in identifying and addressing recruitment challenges across a number of trials [16]. The QRI comprises two phases: phase 1 involves the identification of recruitment issues and phase 2 involves a process of designing and implementing strategies to address the issues identified in phase 1 [15]. The LRRC-QoL study is not a clinical trial and therefore the QRI was modified and used as a framework for the approaches employed during the delivery of the study to drive improvements in recruitment rates, as summarised in Table 1 [15, 16]. Changes to the study recruitment process and study documents were introduced at various timepoints and implemented through substantial and non-substantial amendments to the study ethical approvals. Changes were communicated to research teams via email and videoconference calls. Phases 1 and 2 of the LRRC-QoL ran in tandem to enable response to any additional challenges as they were identified. Screening and recruitment rates were monitored throughout the study duration to identify trends.

**Table 1:** Quintet Recruitment Intervention (QRI)

Quintet Recruitment Intervention (QRI) [15, 16]	Modified QRI
Phase 1 Audio-recording recruitment encounters Interviews with recruiters Mapping recruitment pathways Reviewing trial documentation	Phase 1 Focus group with research nurses: • Microsoft Teams meeting September 2021 • Research teams from all participating UK sites were invited to attend • Aimed to identify reasons for non-participation and to develop strategies to improve recruitment Monitoring central screening and recruitment log: • Facilitated through weekly communications with research teams • Completed using Screened, Eligible, Approached, Randomised (SEAR) framework [17], which was adapted to ‘Recruited’ for this observational study Review of patient-facing study documents through patient and public involvement (PPI) work: • PPI undertaken at two timepoints through individual interviews and a group meeting, all conducted via Microsoft Teams • Patient representatives identified by participating sites and social media posts on Facebook and Instagram cancer support pages. Posts were made by a patient identified by a participating site and who had undergone pelvic exenteration inviting patients with LRRC to participate • The aim of the PPI interviews was to gain a patient-perspective of study processes and recruitment pathways, particularly patient-facing materials • The aim of the PPI meeting was to review the reasons identified for non-participation and identify strategies to improve recruitment
Phase 2 Presenting findings of phase 1 and agreeing ‘plan of action’	Phase 2 Presenting findings to study supervisory team and agreeing strategies

## Results

Overall, 227 patients were recruited across the two workstreams in the LRRC-QoL study from 27 sites in 14 countries. This included 67 patients recruited to workstream I and 160 to workstream II.

### Study setup

The experiences of setting up the LRRC-QoL study are summarised using relevant key areas for consideration described in the ISTT [14].

### Finance and study supplies

International sites were included in the grant application for the LRRC-QoL study with an initial strategy to include four international centres, developing validated translated LRRC-QoL questionnaires in three languages, in addition to English. As the project continued, the number of international centres grew to 20 and 10 languages including English. The study was charity-funded and as such, resources were finite. The most significant expense during the project was professional translation, both during the cross-cultural adaptation of the LRRC-QoL questionnaire and for materials such as patient information leaflets. Finances also presented a challenge given the limited ability to fund clinical collaborators for their time spent working on the study. The funding for the LRRC-QoL study did not include per patient payments; however, the project’s charity funding meant that it was eligible for National Institute for Health Research (NIHR) Clinical Research Network (CRN) portfolio-adoption in the UK. CRN portfolio-adopted studies can benefit from provision for costs including local study trial-coordination and management, data collection, obtaining ethical approval, and the principal investigator’s time [18]. International sites were not able to access these benefits; however, the study funding included allocations for expenses such as postage of the questionnaires, which could be transferred to reimburse participating international sites.

### Contracts, insurance, data ownership, and publishing

From a legal perspective, research or data processing agreements were required between the organisation sponsoring a research study and all participating organisations. In the UK, a document called the Organisation Information Document (OID) for non-commercial studies was used for participating NHS sites [19]; this, however, cannot be used for non-NHS sites. The International Surgical Trials Toolkit describes two options for research agreements with international sites, the first option being to create agreements between the sponsor and each international site. The second option is to create agreements between an international spoke and for the spoke to create contracts with local research sites [20]. In the case of the LRRC-QoL study, the former approach was taken, and an agreement was put in place between the sponsor and each participating international site. The Toolkit describes common areas for disputes with this approach [20], most of which were encountered during the process of putting these agreements in place.

### Issues regarding data sharing agreements

#### Jurisdiction of agreement and governing law

Agreeing the court of jurisdiction that will govern the agreement can be contentious, with parties often preferring that their local or national laws and courts govern the agreement. In the case of the LRRC-QoL study, the preference and original wording of the agreement is that it be governed by English Courts given the study’s English sponsor. Several international sites proposed changes to the agreement, stating that their local laws and courts govern; in all cases, a resolution was reached through remaining silent on jurisdiction. This approach is also advised in the International Surgical Trials Toolkit [20].

#### Warranties, indemnity, and insurance clauses

The LRRC-QoL agreement included a cap on liability of £5000; some sites requested that this be removed given they were not able to cap liability under their indemnity. The sponsor recognised that under English Law, there could not be a cap on liabilities for events such as death or personal injury due to negligence, with the agreement stating that the liabilities will not extend to punitive, indirect, or consequential losses. This strict legislative framework exists to safeguard patients and has evolved following the introduction of key European legislation, the EC directive in 1965 [21]. Though these safeguarding processes are undeniably important, the same strict regulations are applied to questionnaire studies, which pose a much lower risk of harm to patients.

#### International data transfer and brexit

The transfer of personal data is governed by the General Data Protection Regulation (GDPR), implemented in 2018 in European Union (EU) law [22]. The Data Protection Act (DPA) 2018 [23] is the UK’s implementation of GDPR; however, Britain left the EU on the 1st of January 2021, whilst the LRRC-QoL study was underway. Legal advice from the sponsor contract team resulted in the inclusion of Standard Contractual Clauses (SCCs) for the transfer of personal data [24] in data sharing agreements, enabling personal data transfer to continue post-Brexit. On the 28th of June 2021, the EU formally recognised the UK’s data protection standards as adequate [25], meaning SCCs would not be recommended for future studies involving UK and EU sites. However, they are required for countries deemed inadequate by the DPA such as the USA, Canada, and India.

#### Research governance

In the UK, the Health Research Authority (HRA) manages ethical approvals centrally which are granted by Research Ethics Committees (REC). Once HRA and REC approval for a study is in place, Research and Development (R&D) departments for each participating NHS site must grant local approval for the study. The processes for international ethical approvals are variable; for instance, the United States of America (USA) uses an Institutional Review Board (IRB) system where each participating centre requires approval from its own IRB. Overall, more than 40 central and local ethical approvals were required to enable recruitment across 35 sites. The multiple regulatory and ethical approvals required represented one of the main challenges in conducting this international, multi-centre study and reflects existing literature [12, 13].

#### Protocol and data collection

The recruitment strategy for the UK was based on a Patient Identification Centre (PIC) model with the researcher (NM) coordinating quality of life follow-up centrally. This was implemented given the timing of the study during the pandemic to reduce the burden of research activities at a site level as much as possible. The original recruitment strategy involved a two-stage recruitment process with an initial form to consent to contact and a short patient information leaflet followed by a full participation pack including a formal consent form. Online meetings were held with clinicians from each potential international site to understand local processes and care pathways to enable the development of flexible recruitment pathways and to ensure that recruitment targets to enable cross-cultural adaptation of the LRRC-QoL were feasible.

## Modified QRI

### Phase 1: identification of recruitment challenges and strategies

#### Recruitment challenges identified

**Fig. 1:**
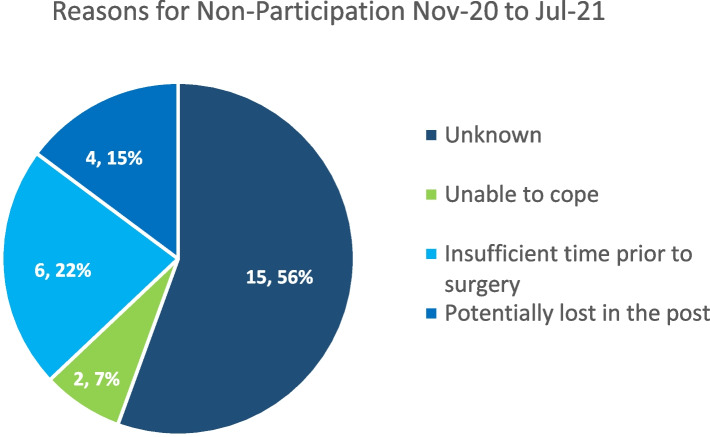
Reasons for non-participation in workstream II November 2020 to July 2021

Review of the central screening and recruitment log within the first 4 months of the study indicated that the two-stage consent process utilised in the UK risked missing the recruitment window between the time of diagnosis/referral to the specialist team and commencing treatment or undergoing surgery. In August 2021, the rate of patients recruited from those approached remained at 20–25%, comparing unfavourably with the 48.1% recruitment rate reported in the original development of the LRRC-QoL in the UK and Australia [26] and other studies regarding QoL in this disease group, reporting rates of around 40% [27, 28]. Figure 1 depicts reasons for non-participation for the 27 patients who did not consent to data sharing out of the 48 patients screened up until July 2021. These included the limited timeframe for recruitment being exceeded (*n* = 6, 22%) and patients feeling unable to cope or ‘*overwhelmed*’ (*n* = 2, 7%). Four (15%) patients reported having returned the participation pack; however, these were never received. For most cases, the reason for non-participation was unknown (*n* = 15, 56%). In terms of the challenges and barriers to recruitment, research teams reported that it was ‘*challenging to get patients to invest in the study when they have not yet met the clinical team*’, particularly in cases where patients had been referred from geographically distant locations and were unable to attend clinic face-to-face due to the distance and the COVID-19 pandemic.

### Strategies identified

#### Recruitment pathways

The research team focus group meeting identified several strategies to improve recruitment, including approaching patients during face-to-face clinic appointments, follow-up telephone contact to prompt patients to complete and return participation packs, and weekly contact from the central research team to prompt sites to screen and approach eligible patients. Clinical teams undertaking verbal consent to share personal contact details with the central research team were also proposed, as a more time-efficient process for patients not being seen face-to-face in clinic. The potential to recruit patients using an online platform was highlighted during PPI work and in site setup discussions.

### Addressing barriers to recruitment

#### Changes to the patient information leaflets (PILs)

The PILs were reviewed during the PPI interviews held in October 2021. Regarding their general appearance, both patients interviewed felt they contained too much information and should be shortened to ‘*the headlines*’. Utilising ‘*text boxes across the page to help draw attention*’ was suggested, in addition to adding the study aims to the front page of all the PILs and using diagrams to help explain the study. Both patients felt the overall explanation of the study was adequate, but more information should be provided in relation to how the study results would be used to affect care. The information related to data management was felt to be excessive, and both patients were in favour of either removing this information or its inclusion as an additional supplement in smaller print.

#### Addressing barriers to recruitment

The previously identified reasons for non-participation were discussed during the PPI group meeting in May 2022. The PPI representatives suggested highlighting the intended benefits of the study in improving patient care and enabling the use of the LRRC-QoL questionnaire in future research. They felt that the ‘*possibility of improving care for patients in the future*’ would be a strong motivating factor to take part in the research, though they acknowledged that it may not be possible to directly address barriers to participation such as patients ‘*finding it “too much” mentally or “overwhelming” around the time of diagnosis with LRRC*’.

#### Study newsletters and collaborative networks

Existing exenterative surgical networks, including the UK Pelvic Exenteration Network (UKPEN) and the PelvEx Collaborative, shared details of the study with their members via email to identify new sites and collaborators. Other multi-centre studies which were open to recruitment during the same period had demonstrated the success of using newsletters circulated via Twitter to generate interest and promote recruitment [29, 30]. This approach was also employed during the delivery of the LRRC-QoL study.

### Phase 2: changes implemented

#### Recruitment pathway and patient information leaflets

A substantial amendment was submitted and approved in March 2021 to remove the two-stage recruitment process, with sites sending potential participants a single participation pack to complete and return directly to the researcher. Online consent via REDCap was also introduced in addition to a study website to enable viewing of the PILs online. The English-language version went live in June 2021; this was followed by Dutch and Danish versions. Additional changes were introduced following the research team focus group meeting, including verbal consent to contact from the central research team and the option to consent and complete patient-reported outcome measures (PROMs) with the researcher via telephone. The PILs were also updated in keeping with the feedback from the PPI work described above. These changes were all approved in an amendment in December 2021.

#### Study newsletter and communications

The first study newsletter was circulated in June 2021 and then at monthly intervals. Regular email communication with sites was maintained throughout the study. The UKPEN and PelvEx Collaboratives sharing details of the study expanded the number of study sites and collaborators from the initial 13 sites to 37 by June 2022.

#### Impact on the study recruitment and retention rates

**Fig. 2:**
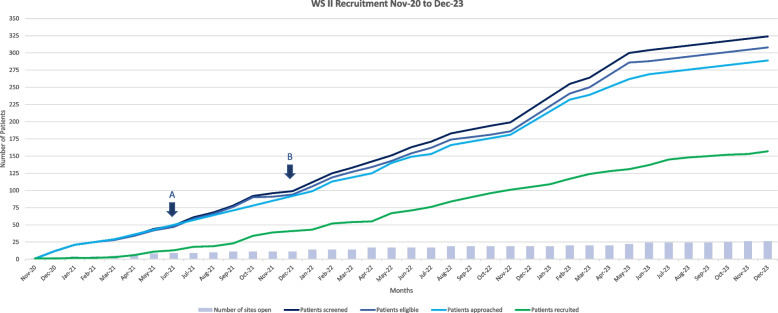
Overall recruitment by month. *Timepoint A indicates the introduction of online consent and participation, timepoint B indicates the introduction of the revised PILs and verbal consent to contact

Workstream II was open to recruitment from November 2020 to December 2023, and recruitment rates are illustrated in Fig. 2. Overall, 311 patients were screened for the study, of which 307 were eligible and 292 approached. Reasons for not approaching eligible patients included high levels of patient distress during consultations resulting in the clinical team feeling it was inappropriate to approach, and not being able to contact patients prior to them commencing treatment, particularly those not receiving treatment at the specialist centre they had been referred to. In total, 160 patients were recruited to workstream II, representing 54.8% of those approached. A recruitment conversion rate of 50% was first achieved in July 2022 and maintained at this level or higher for the remainder of the study. There are several factors which are likely to have contributed, including the addition of two new sites and the conversion of four sites from workstream I to II in early 2022 and the changes to the recruitment process introduced in June and December 2021.

#### Online recruitment

From the introduction of eConsent in June 2021, 73 participants were recruited to the study via REDCap from the UK, the Netherlands, New Zealand, Denmark, and Canada, representing 32.2% of recruitment to the study overall. The ability to complete PROMs online with direct transfer to the central research team was both time-efficient and cost-effective; at international sites in the Netherlands, New Zealand, Denmark, and Canada, this eliminated the need for the local team to collect and transfer PROMs data. Patients who chose to participate online cited reasons including this approach being quicker, easier, and more environmentally friendly. However, online recruitment was not implemented for all sites and languages; teams in the remaining 9 participating countries felt that online recruitment would not be feasible for their local cohort of patients or in line with local ethical approvals. Additionally, it would have been challenging from an implementation perspective to build REDCap surveys in every language.

## Discussion

This work represents one of the first studies to report a detailed exploration of recruitment difficulties in the context of LRRC, identifying several challenges and crucially, strategies to successfully address them. Though a conversion rate from approached to recruited of 50% may seem relatively low, it is higher than those previously reported in PROMs studies of patients with advanced cancer [26–28, 31] and represents a significant achievement in this setting. The barriers to recruitment identified included patients feeling distressed or overwhelmed around the time of diagnosis, similar to those previously described in patients receiving palliative treatment [31]. A lack of previous contact with the clinical team at specialist referral centres could act as a barrier to recruitment.

From a study design perspective, the initial study recruitment process was found to be overly complicated, causing the recruitment window to be missed. Several strategies were identified which successfully addressed these issues. The two-stage recruitment pathway was refined to a more streamlined approach. Utilising verbal consent to contact enabled the recruitment encounter to be undertaken remotely by the central research team. Recruitment rates also improved with the introduction of multiple options for participation, including traditional paper-based methods, online, and via telephone. In relation to patients feeling overwhelmed by the volume of information received both in relation to potential treatments and the LRRC-QoL study, this was addressed by refining the PILs with input from PPI representatives. Many of the challenges encountered during site setup could also apply to multi-centre, international studies across a range of disease settings, including issues related to legal agreements, translation, and multiple ethical approvals. The majority of these issues are highlighted in the International Surgical Trials Toolkit, with guidance to address them [14]. LRRC being a relatively rare form of advanced pelvic malignancy posed additional challenges such as identifying appropriate sites to participate. Existing collaborative networks were harnessed to promote and identify sites, targeting specialist referral centres with a view to maximising recruitment rates.

There is relatively little evidence detailing specific recruitment challenges in LRRC; this is reflected in the small numbers of published trials in this setting, despite LRRC being recognised by both clinicians and patients as an important research area [32]. However, the landscape is rapidly changing, with an increasing focus on improving outcomes in patients with advanced and recurrent colorectal cancer; two important trials are currently underway which are likely to shape future practice regarding pre-operative oncological treatment for LRRC [33, 34]. The GRECCAR 15 trial compares neoadjuvant (induction) chemotherapy followed by re-irradiation with neoadjuvant chemotherapy alone in patients with LRRC who have previously received radiotherapy [33]. The PelvEx II trial compares induction chemotherapy followed by neoadjuvant chemoradiotherapy (including re-irradiation) to neoadjuvant chemoradiotherapy alone [34]. Growing the evidence base regarding recruitment challenges specifically in RCTs in LRRC is likely to take several years; the findings of the LRRC-QoL study could offer important insights for the development of current and future international RCTs, particularly those including HrQoL endpoints.

The utilisation of the QRI to inform this work is a significant strength and led to the identification of effective strategies to improve recruitment in this challenging setting. In relation to the methods applied, it is possible that the central screening and recruitment log was not completely accurate, though regular communications with research teams at sites were maintained to ensure it was as accurate as possible. Regarding PPI, the small numbers of patients participating in this work represents a limitation and reflects the rare nature of LRRC. Additionally, the PPI work and research team focus group meeting were exclusively conducted with English sites and patients, meaning their outcomes are not necessarily generalisable. This decision was undertaken due to the tailored recruitment approaches implemented across international sites, meaning holding a meeting for all research teams may have caused confusion due to the range of approaches applied.

The experience of delivering the LRRC-QoL study has important implications for future work in this disease setting. Utilising the International Surgical Trials Toolkit was highly beneficial in the delivery of the LRRC-QoL study and is likely to benefit future surgical studies and trials navigating this process. Additionally, existing collaborative networks should be harnessed to set up and deliver studies. Undertaking PPI work regarding study delivery processes, and particularly in relation to developing PILs and recruitment strategies, had a resounding positive impact on the study overall. Many funders now stipulate that PPI work is undertaken during the development of research proposals, including NIHR [35]. Moving forward, high-quality PPI work should be considered a routine aspect of the design and implementation process for all clinical studies in LRRC. Though approaches such as studies within a trial (SWATs) are an important instrument in identifying strategies to improve the delivery of clinical trials [36], they are unlikely to be feasible in rare disease settings, such as LRRC. The number of patients required for a SWAT to deliver meaningful results is likely to be impossible to achieve in this context. Future trials in LRRC could incorporate the QRI with a view to confirming and building upon this evidence base.

Regarding PILs, previous work has highlighted the importance of ensuring they are comprehensible, using plain language, and have an attractive layout and structure, with diagrams to support textual information [37]. The LRRC-QoL PPI work echoes these findings as many of the changes to the PILs related to layout and structure. Alternative methods for conveying study information, including multimedia informational videos and illustrations or diagrams, should also be considered. The addition of diagrams to the PILs in the LRRC-QoL study is likely to have contributed to the improvement in recruitment rates. The use of informational videos has been examined through SWATs, which reported that they may help patients to better understand the information being communicated; however, they have not demonstrated a recruitment benefit [38, 39]. Funding was not available to enable the creation of informational videos for the LRRC-QoL study, particularly given the number of languages required. However, this could be explored in future studies.

Developing an understanding of clinical pathways at a site level, particularly in complex disease settings such as LRRC, is imperative to developing recruitment pathways which complement existing processes. The effectiveness of this approach has previously been demonstrated in the context of COVID-19 [40] and is evident in the response to the changes introduced to the LRRC-QoL study recruitment pathway. During the LRRC-QoL study, follow-up was coordinated centrally for most English-speaking sites, in addition to sites in the Netherlands and Denmark, where patients had the option to participate online via REDCap. This approach was primarily implemented to reduce workloads for participating research teams and positively contributed to clinician buy-in, particularly during the pandemic, with reduced availability of research staff support. Overall, this approach had a positive impact on both follow-up retention rates and in gaining feasibility decisions from sites; it should be considered for all future studies involving prospective HrQoL assessments in LRRC.

Finally, offering a variety of methods for completing the LRRC-QoL, including face-to-face, postal, telephone, and online, should be implemented routinely in future studies reporting HrQoL in LRRC. Equivalence across different modes of PROMs administration supports this flexible approach [41]. Furthermore, the ePROM version of the LRRC-QoL was developed in keeping with both EORTC and ISPOR guidance [42, 43]. Undertaking discussions with sites and offering flexibility in the approach to modes of recruitment and follow-up had a significant positive impact on the study. Online consent and PROMs completion enables remote recruitment and follow-up which can offer significant benefits, such as removing the need for face-to-face contact and can be more cost-effective than postal-based methods [44]. The importance and necessity of flexibility in the approach to collecting PROMs data has previously been identified by other research groups working in a similar setting [45]. In the context of the LRRC-QoL study, this facilitated recruitment by ensuring that the approach implemented was appropriate to the local population and participating clinical team.

## Conclusions

Conducting an international, multi-centre prospective cohort study of HrQoL in a relatively rare disease involved many challenges, particularly in relation to recruitment. The recruitment challenges and strategies identified during the delivery of this study provide several recommendations for future work in this field. These include undertaking PPI work during study development, particularly to advise regarding PILs and recruitment strategies, and ensuring flexibility in recruitment and study delivery approaches, particularly at international sites. Other recommendations include partnering with existing collaborative networks where possible and maintaining regular communication with sites, including regular study newsletters. Future collaborative work could be undertaken to identify additional recruitment strategies which are effective in rare disease settings, such as LRRC.

## Data Availability

The datasets generated and/or analysed during the current study are not publicly available due to study follow-up being ongoing but are available from the senior author on reasonable request.

## Abbreviations

CRN: Clinical Research Network
CTRU: Clinical Trials Research Unit
DPA: Data Protection Act
EU: European Union
GDPR: General Data Protection Regulation
HRA: Health Research Authority
HrQoL: Health-related quality of life
IRB: Institutional Review Board
LRRC: Locally recurrent rectal cancer
LRRC-QoL: Locally Recurrent Rectal Cancer—Quality of Life
NIHR: National Institute for Health Research
OID: Organisation Information Document
PIC: Patient Identification Centre
PIL: Patient information leaflet
PPI: Patient and public involvement
PROM: Patient-reported outcome measure
QoL: Quality of life
QRI: Quintet Recruitment Intervention
RCT: Randomised controlled trial
REC: Research Ethics Committees
R&D: Research and Development
SCCs: Standard Contractual Clauses
SEAR: Screened, Eligible, Approached, Randomised
SWAT: Study within a trial
UKPEN: United Kingdom Pelvic Exenteration Network
USA: United States of America
